# A narrative review of the current available literature on the intersection between the climate crisis and paediatric surgical care

**DOI:** 10.1007/s00383-024-05899-3

**Published:** 2024-11-23

**Authors:** Kavita Patel, Anna Ossig, Søren Kudsk-Iversen

**Affiliations:** 1 Barts Health NHS Trust, London, UK; 2 Maastricht University, Maastricht, The Netherlands; 3 Oxford University Hospitals, Oxford, UK

**Keywords:** Climate, Paediatric, Surgery, Syndemic, Health equity

## Abstract

The climate crisis exacerbates health inequities, including in paediatric surgery, creating a vicious cycle. We sought to review (a) existing evidence on the connection between paediatric surgery and climate and (b) how this addresses the vicious cycle. A PubMed search was conducted on 23-08-2023. Articles not commenting on "climate change" and "paediatric surgery" were excluded. Included papers were categorised into emerging themes. Out of 151 search results, seven articles were included. The emerging themes related to "Effect of climate on paediatric surgical illness" (*n* = 3), "Effect of surgery on climate" (*n* = 2), and "Mitigating impact of paediatric surgery" (*n* = 2). Five articles were observational studies, and two were literature reviews, all papers published after 2020. We found limited primary research focusing on the intersection between climate change and paediatric surgery. Articles tend to focus on quantifying impact and mitigation, which does not lend itself to climate justice. The syndemic model of health focuses on the complex interconnections and pathways through which health conditions interact within populations to exacerbate adverse health outcomes. We suggest future research needs to be reframed, with the interconnection between health inequities, the climate crisis, and the wider health system addressed together.

## Introduction

The issue of the climate crisis has been at the forefront of global conversations, including in the context of healthcare. It is well known that worsening climate plays a significant role in health and well-being and continues to negatively impact the burden of disease [1]. We know that health inequity, inequity being different from inequality as it acknowledges the existence of unfair and avoidable differences between populations, leads to marginalised groups disproportionately suffering from the consequences of the worsening climate crisis.

For example, the impacts of air pollution on pulmonary disease and rising temperatures leading to heat-related illnesses are well-established consequences of the climate crisis [2––4]. Furthermore, flooding continues to increase the risk of infectious disease spread, as well as devastating infrastructure and contributing to economic burden [5, 6]. These impacts of the worsening climate lead to a vicious cycle of worsening inequity [7]. To this regard, climate justice is a term that has been used to shift the narrative of climate change away from one that simply focuses on reducing greenhouse gas emissions, and towards an approach that centres around social inequity and the interconnectedness of struggles. Climate justice, therefore, centres the topic of climate change around those marginalised groups that are disproportionately affected by its consequences [8], meaning that solutions are focused on creating a more just world.

Islam and Winkel published an article on climate change and social inequality that offers a conceptual framework demonstrating the link between these two entities [7]. The article outlines how climate change leads to inequality through *exposure* and *susceptibility* to the effects of climate change, and that inequality also affects the *ability to cope and recover* from the impacts of climate change [7]. This links into syndemics research theory, which focuses on disease interactions and population health drivers. Syndemics research was first described by Merrill Singer in relation to substance abuse, violence, and acquired immune deficiency syndrome (AIDS), demonstrating the interdependence of these conditions in exacerbating health outcomes [9]. Singer sought to argue that the human immunodeficiency virus (HIV) epidemic must not be taken in isolation, but that it should be situated within a broader context that is interdependent on the local substance misuse epidemic as well as being exacerbated by structural violence [10]. The syndemics of climate change and worsening health inequity are directly linked with child health and surgical care [11, 12]. Therefore, our aim was to explore how the published literature explains the intersection between the impacts of the climate crisis and paediatric surgical care, and through a narrative review identify themes and possible future implications for paediatric surgical care.

## Method

We used a rapid review strategy and conducted a PubMed search on 23-08-2023, using the following search string: (climate OR planet*) AND (surg* OR periop*) AND (child* OR paed* OR pedi*) AND impact. Articles were eligible for inclusion if they commented on *both* the impact of the climate crisis *and* paediatric surgical care.

Two authors (KP and AO) screened the titles and abstracts to see if articles met the eligibility criteria. Where there was uncertainty, all authors reviewed the paper in question and decision for inclusion was based on agreement following discussion.

Articles meeting the eligibility criteria then went on to a full article review. During this process, data were collected, including on the type of paper (e.g. original research or review), year of publication, first author affiliation, primary/secondary outcomes, and country where data were collected. Additionally, narrative analysis was conducted of the conclusions drawn in the eligible papers to identify any themes linking the climate crisis and health equity.

Due to the nature of the study, no ethical approval was required. We did not undertake any bias or certainty assessments.

## Results

**Fig. 1:**
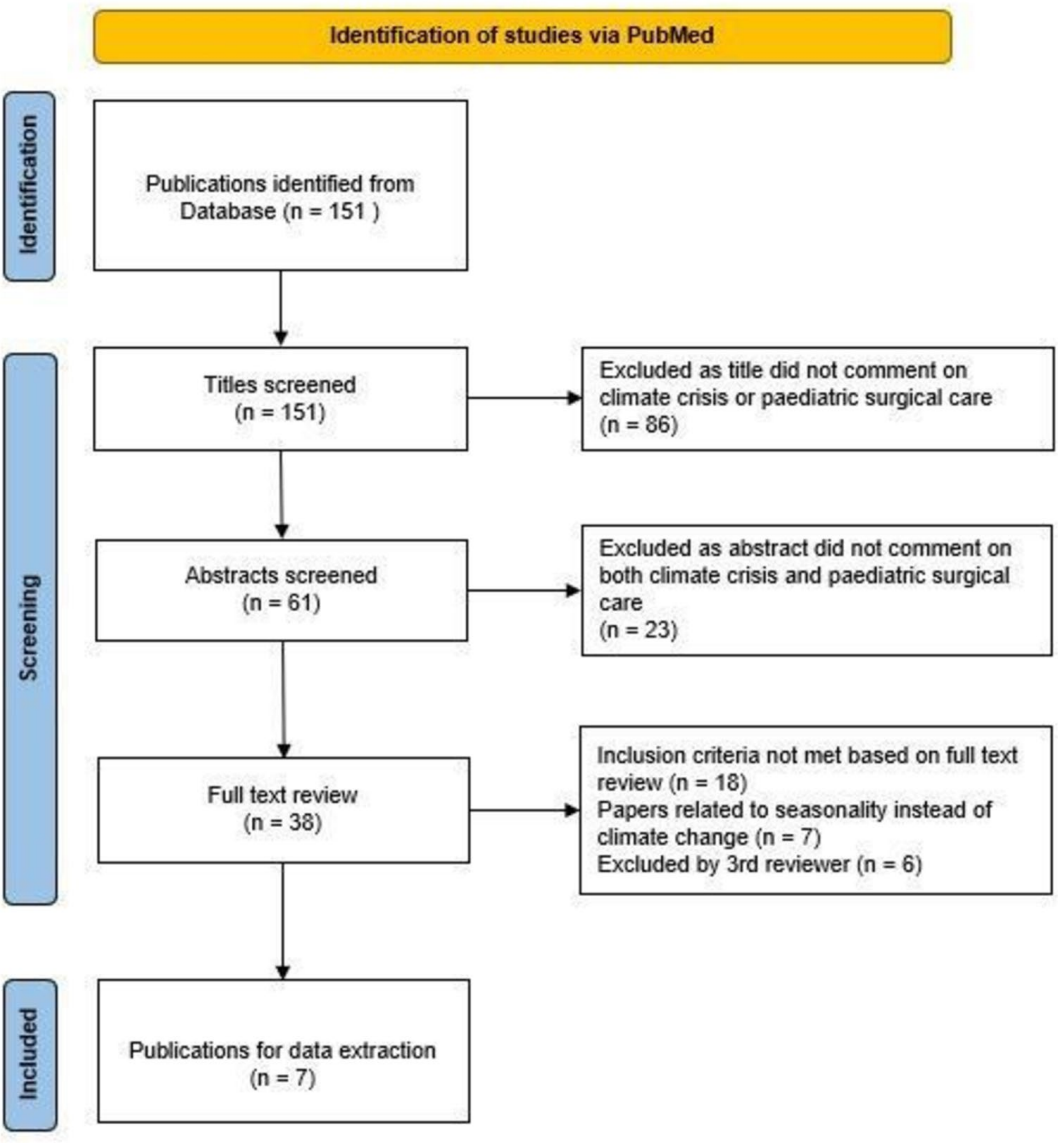
Diagram showing phases of the article review process

The PubMed search results returned 151 articles that were included as part of this review (see Fig. 1). Out of these, seven articles matched the eligibility criteria.

Although we had no restrictions on when the papers were published, eligible articles were all published from 2021 onwards. Five articles were observational studies, and two were literature reviews. We found that all papers had first authors affiliated with institutions in high-income countries, and the five observational studies were all conducted in high-income settings.

In our analysis of the conclusions, the themes that emerged were "Effect of climate change on paediatric surgical illness" (*n* = 3) [8, 13, 14], "Effect of paediatric surgical care on climate change" (*n* = 2) [15, 16], and "Mitigating the impact of paediatric surgical care" (*n* = 2) [17, 18].

Considering the effect of climate change on paediatric surgical illness, Kaufman et al. observed that the estimated kidney stone presentation attributable to heat in South Carolina, USA, was projected to increase due to climate change, with attributable costs [13]. Mun et al. successfully developed prediction models linking air pollution (measured as particulate matter) and climate indicators such as temperature, humidity, solar insolation, wind speed, and greenhouse gases, for example, with the incidence of acute otitis media (AOM) [14]. Increases in AOM was related to decreases in minimum temperature and increased temperature differences and minimum humidity. One prediction model used in this study showed minimum temperature and daylight duration to be the most important variables (i.e. increasing daylight duration led to increases in AOM). Included within this theme was the only paper which commented on both impact of climate on paediatric surgical care and health inequity. In the review by Cockrell et al., the authors discuss the threat of the climate crisis to marginalised groups, including children [8]. They found (a) increased risks of, for example, gastroschisis, spina bifida, and hypospadias due to wildfire exposure and high temperatures; (b) increased risk of preterm birth, which is known to be a risk factor for the need of surgical intervention in the postnatal period, due to severe heat and air pollution; and (c) trauma due to natural disasters to be a common indication for surgical intervention in children. The authors discuss the need for strategies to mitigate the impact of the climate crisis on marginalised groups who are disproportionately affected by the climate crisis, as well as the importance of reducing the impact of healthcare on the climate crisis [8].

The second theme, the effect of paediatric surgical care on climate change, was described by Hansen et al. [16]. Their observational study looked at the use of volatile gases in paediatric anaesthesia and used this to calculate carbon emissions from operating rooms in Seattle Children’s Hospital, USA. The paper also discusses methods for reducing carbon dioxide (CO_2_) emissions in this setting by removing desflurane as an anaesthetic agent and reducing the default fresh gas flow rate on anaesthetic machines [15]. The observational study by Chua et al. [17] describes the specific sources of carbon emissions and associated estimates of carbon emissions from the interventional radiology department at a hospital in New York, USA [16]. The highest estimates of CO_2_ emissions in this setting were related to indoor climate control systems that use substantial amounts of electricity and gas to power the unit’s climate control system/heating, ventilation, and air-conditioning system. Interestingly, anaesthetic gases contributed the least to CO_2_ emissions from this department.

The final theme, mitigating the impact of paediatric surgical care, was described by Cockrell et al. [18]. They found that the incorporation of telehealth into paediatric surgical and pre-anaesthesia clinics resulted in significant CO_2_ emission reductions, estimated at 887,006 patient-miles saved and 688,317 fewer pounds of CO_2_ emitted [17]. A narrative review by Yates et al. [19] summarised existing research on sustainable surgical practices to enable surgeons, anaesthetists, and obstetricians to advocate for improved environmental sustainability practices [18]. The authors discuss improvements in infrastructure and equipment such as adjustments in purchasing strategies of surgical equipment to include environmentally electronic devices, mercury-free purchasing, and recycled products. Additionally, they discuss changes in patient care to reduce the use of volatile anaesthetics and utilise telehealth visits instead of clinic visits where possible.

**Fig. 2:**
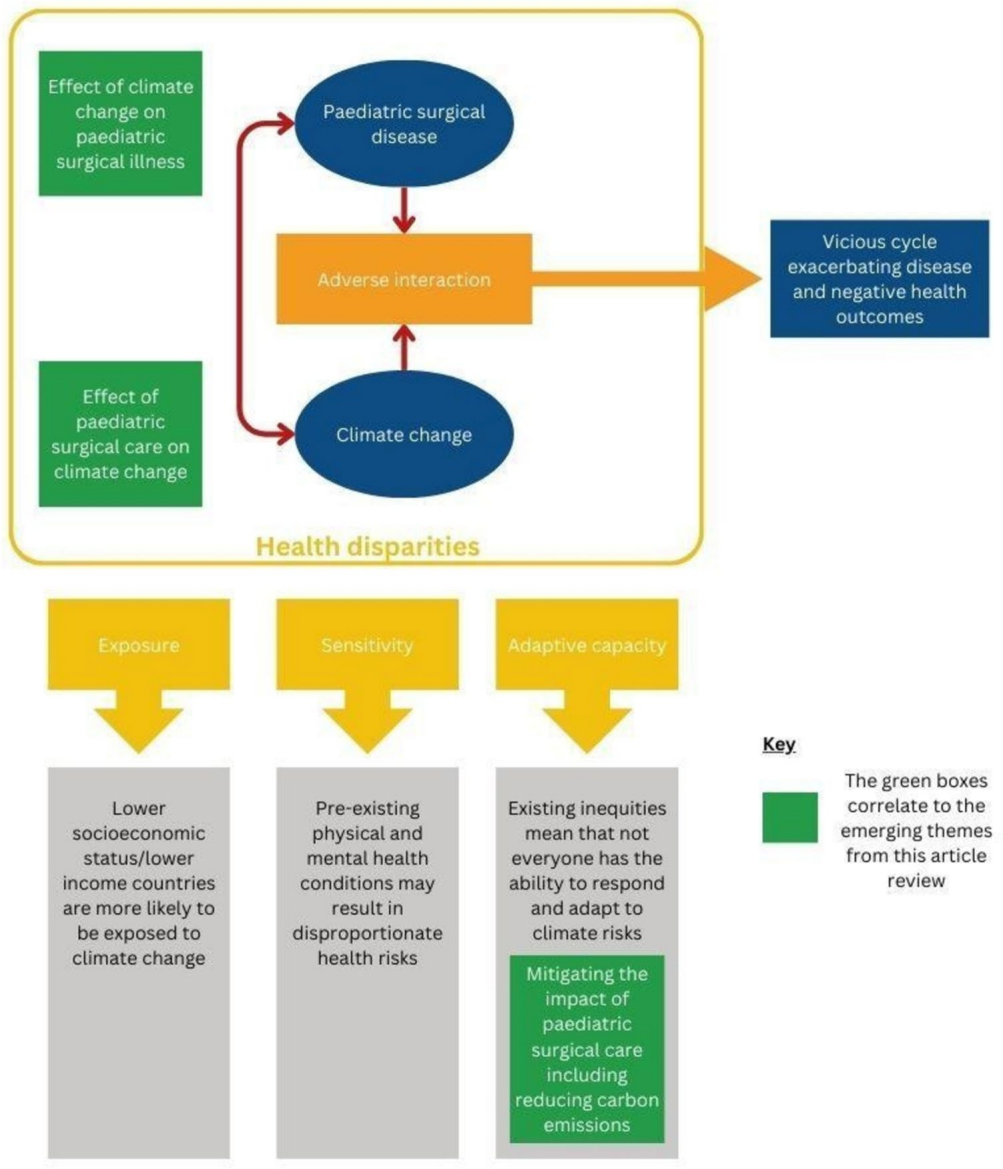
Syndemic model demonstrating the vicious cycle of climate change and paediatric surgery. Adapted from an original work by Islam and Winkel [7] and The Public Health Agency of Canada [19]

To visualise how the emerging themes identified relate to the vicious cycles of the climate crisis and health inequity, we developed Fig. 2. Here, the green boxes in the diagram relate to the emerging themes that the articles were categorised into. As demonstrated, all of the included articles either focus on climate change as a direct cause of surgical illness (e.g. increases in kidney stone presentations [13], the link between air pollution and acute otitis media [14]) or paediatric surgery as a contributor to climate change (e.g. focus on carbon counting [15, 16]) or mitigating the impact of paediatric surgery through reduction of carbon emissions [17, 18].

## Discussion

We found limited primary research focusing on the intersection between climate change and paediatric surgery.

In Fig. 2, we linked “measures to reduce carbon emissions” to “*adaptive capacity*”, to show that reducing carbon emissions is a part of the process of adaptability. However, efforts to reduce carbon emissions form only a small part of “*adaptive capacity*”, and existing inequality means that not everyone is able to respond and adapt to climate risks in the same way. Figure 2 also demonstrates that research relating to *exposure* and *sensitivity* to climate change in the context of paediatric surgical care was absent in the articles reviewed.

Of the articles included in this review, there was a focus of research on the impact of paediatric surgery and anaesthesia as a contributor to climate change. Whilst it is important to quantify healthcare’s environmentally unsustainable culture, there is a lack of discussion on how this is contrary to the Hippocratic Oath which states that doctors should “do no harm”. To elaborate, efforts to reduce healthcare carbon emissions alone may be perceived as simply tokenistic, if they are not matched by cultural changes within medicine that address pre-existing structural discrimination, for example racism, sexism, ableism, and socio-economic status [20]. Steps towards creating a more environmentally sustainable healthcare culture therefore requires an intersectionality perspective. An intersectionality perspective on the climate crisis considers the impact of inequalities together, rather than siloed approaches that look at climate change, health, and discrimination separately [21, 22].

It was interesting to note that all articles in this review were from higher-income countries, with a focus on quantifying carbon impact and mitigation, which does not itself lend itself to climate justice. The impact of the climate crisis varies between countries, with national climate policies most likely aligning with their relative contribution to global CO_2_ emissions [22], and their preparedness and vulnerability to the effects of climate damage [23]. In other words, wealthy nations with large historical emissions are more likely to aim for mitigation, while countries already feeling the effects of climate change, predominantly countries with already stretched resources, might focus more on adaptation and climate reparations. This is reflected in the UN’s Environmental Programme Emissions Gap Report which states that unprecedented action is required and** “**For high-income countries, this implies further accelerating domestic emissions reductions, committing to reaching net zero as soon as possible … For low- and middle-income countries, it means that pressing development needs must be met” [24, p. XVI]. This focus may drive research funding and research priorities, and with that in high-income countries there may be a skew towards mitigation and quantifying impact, rather than topics of climate justice. This could therefore explain our findings, in view of the predominance of papers originating from high-income countries. The *transition* towards a more sustainable future must also be done with justice, which means ensuring that those that are the most vulnerable are involved at every step of the decision-making process. This must also be the case when carrying out research. Without ensuring that research is representative across the globe, it will only act to perpetuate the vicious cycle of inequality and shift the focus towards the needs and expectations of high-income countries.

As the demand for paediatric surgical care increases globally, understanding the intersection between paediatric surgical care and climate justice will be crucial for the development of policies. This review identifies a gap in current research, which lacks a focus on addressing the vicious cycle or placing intersectionality at the heart of research on the climate crisis and paediatric surgical care. This calls for more comprehensive and cohesive research into the subject. With more comprehensive research, policies may be able to better align with the issue of reducing inequality and take steps towards climate justice. The fact that all articles in this review were published after 2020, hopefully reflects an increase in interest in this topic over recent years and acknowledges the call for healthcare to recognise its vital role in achieving climate justice.

The limitations of this review are that this was a rapid review with focus on only one database. The search is likely missing out on papers from low- and middle-income countries. While it would be worth carrying out a wider search using multiple databases, the findings of this review are still relevant. Furthermore, due to there being a single reviewer for each article title and abstract, there may have been bias based on opinions and interpretation of the reviewer. This was, however, limited by frequent meetings and discussion beforehand about the study focus and definitions.

## Conclusion

By overlooking the vicious cycle, we risk reducing the solution to the climate crisis as simply being a matter of carbon counting. The syndemic model of climate change and paediatric surgery demonstrates the broader interconnections and pathways through which health conditions interact within populations to exacerbate adverse health outcomes [20, 25]. We suggest that future research should be reframed, addressing the intersection between health inequities, the climate crisis, and the wider health system together if we are to make progress towards achieving climate justice.

## Data Availability

No datasets were generated or analysed during the current study.

## References

[1] Landrigan PJ, Fuller R, Acosta NJR et al (2018) The Lancet Commission on pollution and health. Lancet 391:462–512. 10.1016/S0140-6736(17)32345-0 29056410 10.1016/S0140-6736(17)32345-0

[2] World Health Organization. The Global Health Observatory Indicator 3.9.1: mortality rate attributed to household and ambient air pollution (per 100 000 population). Available at: https://www.who.int/data/gho/data/indicators/indicator-details/GHO/ambient-and-household-air-pollution-attributable-death-rate-(per-100-000-population). Accessed on 25th May 2024.

[3] Figueres C, Landrigan PJ, Fuller R (2018) Tackling air pollution, climate change, and NCDs: time to pull together. Lancet 392:1502–1503 30496047 10.1016/S0140-6736(18)32740-5

[4] World Health Organization (2011) Public Health advice on preventing health effects of heat

[5] Watts J (2014) Haiti making good progress in health but challenges remain. Lancet 384(9952):1413–1414. 10.1016/S0140-6736(14)61835-3 25390310 10.1016/S0140-6736(14)61835-3

[6] Keellings D, Hernández Ayala JJ (2019) Extreme rainfall associated with Hurricane Maria over Puerto Rico and its connections to climate variability and change. Geophys Res Lett 465:2964–2973

[7] Islam M, Winkel J (2017) Climate change and social inequality. DESA Working Paper No.152. 10.18356/a39cdf00-en

[8] Cockrell HC, Hansen EE, Gow K, Fecteau A, Greenberg SLM (2023) The intersection of pediatric surgery, climate change, and equity. J Pediatr Surg 58(5):943–948 36792419 10.1016/j.jpedsurg.2023.01.017

[9] Singer MC (1994) Aids and the health crisis of the U.S. urban poor: the perspective of critical medical anthropology. Soc Sci Med 39:931–948 7992126 10.1016/0277-9536(94)90205-4

[10] Mendenhall E, Kohrt BA, Logie CH et al (2022) Syndemics and clinical science. Nat Med 28:1359–1362. 10.1038/s41591-022-01888-y 35864249 10.1038/s41591-022-01888-y

[11] Lee EE, Stewart B, Zha YA, Groen TA, Burkle Jr FM, Kushner AL (2016) Surgical care required for populations affected by climate-related natural disasters: a global estimation. PLoS Currents 10:8. https://www.ncbi.nlm.nih.gov/pmc/articles/PMC4999354/10.1371/currents.dis.e601960a8cd66c3083d160877abfdde4 PMC4999354 27617165

[12] Unicef. The Children’s Climate Risk Index (CCRI). Interactive Atlas (beta version, Aug 2021). Available at: https://experience.arcgis.com/experience/0d9d2209bf104584a65e012b03b6d3f8/#data_s=id%3AdataSource_2-17b3a7be4c5-layer-1_427%3A203. Accessed on 15th Aug 2024

[13] Kaufman J, Vicedo-Cabrera AM, Tam V, Song L, Coffel E, Tasian G (2022) The impact of heat on kidney stone presentations in South Carolina under two climate change scenarios. Sci Rep 12(1):369. 10.1038/s41598-021-04251-2 35013464 10.1038/s41598-021-04251-2 PMC8748744

[14] Mun SK, Chang M (2022) Development of prediction models for the incidence of pediatric acute otitis media using Poisson regression analysis and XGBoost. Environ Sci Pollut Res Int 29(13):18629–18640. 10.1007/s11356-021-17135-9 34694557 10.1007/s11356-021-17135-9

[15] Hansen EE, Chiem JL, Righter-Foss K, Zha Y, Cockrell HC, Greenberg SLM, Low DK, Martin LD (2023) Project SPRUCE: saving our planet by reducing carbon emissions, a pediatric anesthesia sustainability quality improvement initiative. Anesthesia Analgesia 137(1):98–107. 10.1213/ANE.0000000000006421 37145976 10.1213/ANE.0000000000006421

[16] Chua ALB, Amin R, Zhang J, Thiel CL, Gross JS (2021) The environmental impact of interventional radiology: an evaluation of greenhouse gas emissions from an academic interventional radiology practice. J Vasc Interv Radiol 32(6):907-915.e3. 10.1016/j.jvir.2021.03.531 33794372 10.1016/j.jvir.2021.03.531

[17] Cockrell HC, Maine RG, Hansen EE, Mehta K, Salazar DR, Stewart BT, Greenberg SLM (2022) Environmental impact of telehealth use for pediatric surgery. J Pediatr Surg 57(12):865–869. 10.1016/j.jpedsurg.2022.06.023 35918239 10.1016/j.jpedsurg.2022.06.023

[18] Yates EF, Bowder AN, Roa L, Velin L, Goodman AS, Nguyen LL, McClain CD, Meara JG, Cooper Z (2021) Empowering surgeons, anesthesiologists, and obstetricians to incorporate environmental sustainability in the operating room. Ann Surg 273(6):1108–1114. 10.1097/SLA.0000000000004755 33630452 10.1097/SLA.0000000000004755

[19] Public Health Agency of Canada (2022) Chief Public Health Officer of Canada’s Report on the State of Public Health in Canada 2022: mobilizing public health action on climate change in Canada. Public Health Agency of Canada, Ottawa

[20] Deivanayagam TA et al (2023) Envisioning environmental equity: climate change, health, and racial justice. Lancet 402(10395):64–78. 10.1016/S0140-6736(23)00919-4 37263280 10.1016/S0140-6736(23)00919-4 PMC10415673

[21] Wortzel JR, Guerrero APS, Aggarwal R, Coverdale J, Brenner AM (2022) Climate change and the professional obligation to socialize physicians and trainees into an environmentally sustainable medical culture. Acad Psychiatry 46(5):556–561. 10.1007/s40596-022-01688-z 35879599 10.1007/s40596-022-01688-z PMC9312321

[22] Our World in Data. CO_2_ emissions. Available at: https://ourworldindata.org/co2-emissions. Accessed on 29th May 2024

[23] Notre Dame Global Adaptation Initiative. Available at: https://gain.nd.edu/our-work/country-index/. Accessed on 29th May 2024

[24] United Nations Environment Programme (2023) Emissions Gap Report 2023: broken record—temperatures hit new highs, yet world fails to cut emissions (again). Nairobi. 10.59117/20.500.11822/43922.

[25] Singer M, Bulled N, Ostrach B, Mendenhall E (2017) Syndemics and the biosocial conception of health. Lancet 389(10072):941–950. 10.1016/S0140-6736(17)30003-X 28271845 10.1016/S0140-6736(17)30003-X

